# Social media use and health promoting lifestyle: an exploration among Indian nursing students

**DOI:** 10.17533/udea.iee.v38n2e12

**Published:** 2020-07-10

**Authors:** Maya Sahu, Sailaxmi Gandhi, Manoj Kumar Sharma, P. Marimuthu

**Affiliations:** 1 RN, RM, BSN, MSN. Ph.D Scholar. Department of Nursing, National Institute of Mental Health & Neurosciences(NIMHANS) (Institute of National Importance), India. Email: mayamonsahu@gmail.com National Institute Of Mental Health And Neurosciences National Institute of Mental Health & Neurosciences India mayamonsahu@gmail.com; 2 RN, RM, BSN, MSN, Ph.D. Additional Professor & Head, Department of Nursing, National Institute of Mental Health & Neurosciences. NIMHANS. India Email: sailaxmi63@yahoo.com. Corresponding author National Institute Of Mental Health And Neurosciences National Institute of Mental Health & Neurosciences India sailaxmi63@yahoo.com; 3 M. Phil (Medical & Social Psychology), Ph.D. Professor, Department of Clinical Psychology, National Institute of Mental Health & Neurosciences NIMHANS. India Email: shutclinic@gmail.com National Institute Of Mental Health And Neurosciences National Institute of Mental Health & Neurosciences India shutclinic@gmail.com; 4 B.Sc, M.Sc, Ph.D. Department of Biostatistics, National Institute of Mental Health & Neurosciences. NIMHANS. India Email: p_marimuthu@hotmail.com National Institute Of Mental Health And Neurosciences National Institute of Mental Health & Neurosciences India p_marimuthu@hotmail.com

**Keywords:** social media, health promotion, healthy lifestyle, students, nursing, cross-sectional studies., medios de comunicación sociales, promoción de la salud, estilo de vida saludable, estudiantes de enfermería, estudios transversales., mídias sociais, promoção da saúde, estilo de vida saudável, estudantes de enfermagem, estudos transversais.

## Abstract

**Objective.:**

To evaluate the use of social media and the health promoting lifestyle profile of indian nursing students.

**Methods.:**

A cross-sectional study was carried out with 125 students (89 undergraduate and 36 graduate) from various Nursing universities in India, who provided information on their sociodemographic data, the Bergen Social Media Addiction Scale (BSMAS) and the Health Promoting Lifestyle Profile (HPLP-II).

**Results.:**

Regarding the BSMAS, the participants had an average of 12.8 (maximum possible = 30); 42% reported they frequently delayed their sleep due to using social media; 9% had excessive use of social media; by gender, men had higher total score than women. With respect to the health promoting lifestyle profile, the total average was 126.9 (maximum possible = 208); no difference was observed by gender in the total score and men scored better in the domain of physical activity; students living with their families had higher scores in the domain of health responsibility than those living alone; and graduate students had better scores than undergraduate students in the scale total and in the domains of health responsibility, spiritual relations, and interpersonal relations.

**Conclusion:**

There is excessive use of social media, especially among male students. This study also revealed lower scores than those desired in the domains of Health Promoting Lifestyle, especially for physical activity, health responsibility, stress management, and nutrition. Thereby, the findings may be used to improve health literacy on social media, as well as promote a positive lifestyle among nursing students.

## Resumen

**Objetivo.:**

Evaluar el uso de las redes sociales y el estilo de vida promotores de salud los estudiantes de enfermería hindúes.

**Métodos.:**

Se hizo un estudio de corte transversal con la participación de 125 estudiantes (89 de pregrado y 36 de posgrado) de varias universidades de enfermería en India brindaron información sobre datos sociodemográficos, la Escala de adicción a las redes sociales de Bergen (BSMAS) y el Perfil de estilos de vida promotores de salud (HPLP-II).

**Resultados.:**

Con respecto al BSMAS los participantes tuvieron un promedio de 12.8 (máximo posible = 30); un 42% reportó que con frecuencia tenía retraso en el sueño debido al uso de las redes sociales; un 9% tiene uso excesivo de las redes sociales; por sexo, los hombres tuvieron mayor puntaje total que las mujeres. Pasando al Perfil de estilos de vida promotores de salud -HPLP-II- el promedio total fue de 126.9 (Máximo posible = 208); no se observó diferencia por sexo en el puntaje total y los hombres tuvieron mejor puntaje en el dominio de actividad física; los estudiantes que residen con su familia tienen mayor puntaje en el dominio de responsabilidad en salud que los que viven solos; y los estudiantes de posgrado tienen mejores puntajes que los de pregrado en el total de la escala y en los dominios de responsabilidad de salud, las relaciones espirituales y las relaciones interpersonales.

**Conclusión.:**

Hay un uso excesivo de las redes sociales, especialmente entre los estudiantes varones. Este estudio también reveló puntuaciones más bajas de las deseadas en los dominios de estilos de vida promotores de la salud, especialmente para la actividad física, la responsabilidad de la salud, el manejo del estrés y la nutrición. Por lo tanto, los hallazgos se pueden utilizar para mejorar la alfabetización en salud en las redes sociales, así como para promover un estilo de vida positivo entre los estudiantes de enfermería.

## Resumo.

**Objetivo.:**

Avaliar o uso de redes sociais e promotores de saúde no estilo de vida de estudantes de enfermagem hindus.

**Métodos.:**

Foi realizado um estudo transversal com a participação de 125 estudantes (89 de graduação e 36 de pós-graduação) de várias universidades de enfermagem da Índia, que forneceram informações sobre dados sociodemográficos, a Escala de Adição de redes sociais Bergen (BSMAS) e o Perfil dos promotores de saúde no estilo de vida (HPLP-II).

**Resultados.:**

Em relação à Escala de Dependência em Redes Sociais - BSMAS - os participantes tiveram uma média de 12.8 (máximo possível = 30); 42% relataram que frequentemente tiveram atraso no sono devido ao uso de redes sociais; 9% fazem uso excessivo de redes sociais; por sexo, os homens tiveram uma pontuação total mais alta que as mulheres. Indo para o Perfil de Estilo de Vida dos Promotores de Saúde - HPLP-II -, a média total foi de 126.9 (Máximo possível = 208); nenhuma diferença foi observada por sexo na pontuação total e os homens tiveram melhor pontuação no domínio da atividade física; os estudantes que residem com suas famílias obtêm pontuação mais alta no domínio da responsabilidade em saúde do que os que moram sozinhos; e os estudantes de pós-graduação obtêm uma pontuação melhor do que os graduados na escala geral e nos domínios de responsabilidade com a saúde, relacionamentos espirituais e relacionamentos interpessoais.

**Conclusão.:**

Há um uso excessivo de redes sociais, especialmente entre estudantes do sexo masculino. Este estudo também revelou pontuações mais baixas do que o desejado nos domínios do estilo de vida que promovem a saúde, especialmente em atividade física, responsabilidade em saúde, controle do estresse e nutrição. Portanto, os resultados podem ser usados para melhorar a alfabetização em saúde nas redes sociais, bem como para promover um estilo de vida positivo entre os estudantes de enfermagem.

## Introduction

With technology advancement, social media use recently has become immensely popular leisure activities among adolescents.(1) Different social media such as *Whatsapp, Facebook, Twitter, Instagram, Youtube* etc. provide a common place where individuals own their account/profile and connect with people all over the world.(2,3) They get access to it by logging into their account from anywhere and anytime using smart phone, laptop or desktop. They interact with others by sharing their thoughts, pictures, videos and creative ideas through posts or blogs.(4,5) It is a wonderful platform of being praised or getting opinions from hundreds of real-life or virtual friends which can either boost the morale or down it as well.(6) Further social media gives the opportunity of meeting friends based on shared interest, chatting, mailing, playing games and gambling.(7,8)

Social media has been found to be used for various other purposes among medical, nursing or other health care students. A study conducted among medical students found entertainment, staying updated, socializing and academic purposes to be the main reasons for its use,(9) whereas another study mentioned its use for health related information.(10) Engagement with social media can be personal, professional, or both. A study from Turkey reported that 89.3% of the participants use social media for professional purposes.(11)

Social media was also found to be helpful in modification of health related behaviour.(12) Use of social media has bi-directional effect; for some it is a way of relaxation whereas for some it increases stress. Similarly, different terminologies were used over the studies, such as ‘*technostress*’, referring stress due to overuse of information and communication technologies.(13) In addition to that, negative effects like ‘*social overload,*’(14) relentlessness of ‘*always on*’ culture(15) and so on are on rise.

With the paradigm shift from medical to a public health perspective in mental health, physical and mental health promotion is an emerging field when considering the importance of holistic health. There is an alarming overgrowth in non-communicable diseases and mental health issues in India.(16,17) This can be addressed through encouraging individual responsibility towards adopting healthy life style considering that health promoting lifestyle plays a major role to promote both physical and mental health.(18) 

According to WHO, healthy behaviour helps one be healthy as well as not have diseases.(19) In nursing, health promotion is considered to be an important concept. As they provide health education to the individuals, families and the communities, nurses are often expected to be role models of health promoting lifestyles for them. Nursing students are future health professionals who will activate the common people towards health promotion.(16) So, it is important to ensure healthy lifestyle among nursing students. Many studies conducted in US and European countries, showed that university students were less involved in health-promoting behaviors.(20-22) Out of the domains of health promoting behaviour, healthy diet and physical activity were the most neglected domains. A study from Iran which was conducted among 500 students found lowest score in nutrition and physical activity.(20) Similarly, another study from Jordan which was conducted among 167 nursing students reported physical activity to be the lowest scored domain.(22) A study from Kuwait documented higher scores in spiritual growth and stress management for older students.(23) A few studies from India also found lowest scores for diet and exercise and showed age and gender differences in regard of health promoting lifestyle.(16,24) 

For nursing students who have to stay in hostel, it may be exciting for the first time being away from home and taking on independent roles but for many, it brings new challenges, such as managing their finances, shopping and food preparation. Thus, focus may not be on healthier diet rather they prefer to opt for fast food, packet food or instant preparation of food. Other challenges might be time management, a lack of structure; which can lead to boredom, coping with loneliness.(25) These may navigate them to excessive use of internet or being online or available in social media. Their procrastination, laziness or stress of being always online may lead them not choosing a healthy lifestyle in terms of reduced physical activity or health responsibility. Besides, their young age makes them believe that they do not have much health problems which can lead to negligence of health promoting lifestyle. 

Social media use have been found to be more among particular groups especially the youth and Social Network Site addiction is associated with impaired health and well-being.(15,26) Studies conducted to find out the usage pattern among nursing students in India are less and there is lack of empirical data for effect of social media on health promoting lifestyle which leads to the present study. The study aims to assess the social media use and health promoting lifestyle among nursing students. It also attempts to find out association between socio-demographic variables and their social media use as well as lifestyle behavior.

## Methods

Study design. A cross-sectional descriptive study design was followed. Based on convenience sampling and their willingness, 125 undergraduate and postgraduate nursing students from various colleges of nursing in India who came for clinical experience in the year 2017-2018, took part in the study. 

Instruments*. (i) Socio-demographic data sheet*. It was prepared by the researchers and included socio-demographic details, internet use details, biological functions, offline activities and substance use history of the students. Few open ended questions were to explore their perception about effect of social media on promoting health; *(ii) Bergen Social Media Addiction Scale (BSMAS); (iii)* It is a 5-point Likert scale adapted from Bergen Facebook Addiction Scale (BFAS).(27) It has six items where score ranges for each item from 1-5 with very rarely as 1 and very often as 5, yielding a total minimum score of 6 to maximum 30. The scale is composed based on the addiction symptoms such as salience, mood modification, tolerance, withdrawal symptoms, conflict, and relapse.(4) Higher the score in BSMAS, stronger the addiction to social media, and a total score more than 19 reflects the user is at-risk of developing problematic social media use;(2) *(iv)Health Promoting Lifestyle Profile II (HPLP II):* It is a 4-point Likert scale with 52 items including six sub-scales: *health responsibility*, *physical activity*, *nutrition*, *spiritual growth*, *interpersonal relationships*, and *stress management.*(28) The author recommended to use mean of the sub-scale rather than the total scores. The higher score in HPLP‑II shows the higher level of health‑promoting behavior. The psychometrics of the tool is established(29,30) and it has been used in Indian settings too.(16)

Procedure*.* Permission was obtained from the respective college Principals or teachers. The subjects were explained briefly about the study and invited to participate. Informed consent was obtained from them. Both male and female students were included. They were seated in a room and data were collected by the first author who also clarified their queries. It took approximately 30 minutes to complete the questionnaire.

Ethical issues. The study obtained ethical clearance from Institute Ethics Committee. The participants were briefed about the study aim and objectives and requested to participate in the study. Written informed consent was obtained from all the participants and they were given freedom to withdraw from the study whenever they wanted. Confidentiality of the participants was assured as the data collection tools did not include any identifying information. Codes were used rather than names to ensure anonymity. Probes were used for eliciting response from each participant.

Data analysis*.* Data were analyzed using Statistical Package for Social Science (IBM Statistics SPSS 22) at a significance level of 0.05. Descriptive statistics were used for presenting the baseline profile of the participants and inferential statistics such as Mann-Whitney test was done to find out the association of health promoting lifestyle and social media use with socio-demographic variables. For the qualitative part, content analysis was done and data were presented. 

## Results

Sample characteristics*.* Mean age of the 125 subjects was 25.25 years (SD= 4.76). Majority of them were female (86.4%) and undergraduate nursing students (71.2%). Around 93% were single (the remaining 7% were married), 5.6% had children, 79% were from nuclear family and 70% were staying in hostel. Four percent of the sample had history of substance use (Table 1). 

**Table 1:** Socio-demographic variables

Variables	Category	Frequency (%)
Gender	Female	108 (86.4)
Gender	Male	17 (13.6)
Education	Under-graduate	89 (71.2)
Education	Post-graduate	36 (28.8)
Marital status	Single	116 (92.8)
Marital status	Married	9 (7.2)
Family type	Nuclear	99 (79.2)
Family type	Joint	26 (20.8)
Residence	Family	38 (30.4)
Residence	Hostel	87 (69.6)
Children	Yes	7 (5.6)
Children	No	118 (94.4)
Substance use	Yes	5 (4)
Substance use	No	120 (96)

Current internet and social media use in the sample*.* Around 97.6% said that they had access to internet and most commonly used device was mobile phone (84%), followed by laptop (4.8%). Eleven percent of the sample reported that they used both devices for accessing internet. Out of the participants who reported current internet and social media use, mean age of initiation of mobile phone was 17.02 years (SD=2.96) whereas access to internet was at the age of 16.8 years (SD=3.11). Mean hours of usage of internet for recreation purpose was 2.17 hours (SD=1.85) which ranged from 1 hour to as long as 14 hours per day. Most commonly used application was Whatsapp among the social media, (97.5%) followed by watching videos (86.1%), listening to songs (85.2%), other social networking sites (80.3%), email (66.4%), online shopping (29.5%) and pornography (4.1%). Eighty eight percent reported that they used more than one social media at a time.

Effect on sleep and other offline activities*.* Nursing students’ mean hours of sleep at night was 6.64 (SD=1.13), which ranged from 4 hours to 9 hours. Social media use was related to frequent delay in sleeping for 42% of the sample. Sleep was delayed by 1 hour for 34.4%, 1-2 hours for 12% and more than 2 hours for 5.6% of the sample. Majority of the participants (88%) pursued some kind of hobby. Most common hobbies were playing games or doing exercise (25.6%) followed by dancing, singing or listening to music (20%), reading books (12%), cooking or gardening and watching TV (12%), talking with family and friends (12%) and drawing or writing poems (5.6%). 

Perception regarding effect of social media on health promotion*.* Majority (67.2%) of the participants said that they shared messages on positive health regularly on social media whereas 32.8% did not. More than half (54.1%) said that they did not share any messages on importance of physical activity while majority of them expressed that they were happy to share messages on diet (61%), spirituality (54.9%) and inter-personal relationship (65.6%). Around 51% perceived that their friends also reacted positively for sharing these messages and they felt good about it. Overall, 85.2% of the participants felt that use of social media and especially sharing these messages promoted their life positively though 14.8% of them did not agree to same. 

Social media addiction. Mean score of BSMAS was 12.79±5.4 which ranged from 6 to as high as 30. Table 2 shows item-wise score of BSMAS. Around 9% scored over 19 indicating that a significant portion may develop overuse of social media. Proportionately more percentage of students said that they felt using it excessively (13.2%), it helped them to overcome personal problems (27.9%), attempted to reduce its use but went in vain (27.9%). Around 24% used it to the extent that it had a negative impact on their studies.

**Table 2:** Item-wise BSMAS score

Items	Very rarely *n* (%)	Rarely *n* (%)	Sometime *n* (%)	Often *n* (%)	Very often *n* (%)
Item 1 “…spent a lot of time thinking about social media”	62 (50.8)	29 (23.8)	22 (18.0)	5 (4.1)	4 (3.3)
Item 2 “… felt an urge to use social media more and more”	48 (39.3)	26 (21.3)	32 (26.2)	13 (10.7)	3 (2.5)
Item 3 “… used social media to forget about personal problems”	40 (32.8)	22 (18.0)	26 (21.3)	23 (18.9)	11 (9.0)
Item 4 “… tried to cut down on the use of social media without success”	56 (45.9)	26 (21.3)	24 (19.7)	8 (6.6)	8 (6.6)
Item 5 “… become restless or troubled if you have been prohibited from using social media”	62 (50.8)	20 (16.4)	25 (20.5)	9 (7.4)	6 (4.9)
Item 6 “… used social media so much that it has had a negative impact on your job/studies”	45 (36.9)	24 (19.7)	24 (19.7)	17 (13.9)	12 (9.8)

Health promoting lifestyle*.*Table 3 showed that the mean total HPLP-II score was 126.92±19.46 (range 81-171). It was observed that higher mean scores in the subscales of HPLP II were for inter personal relationship (25.73± 4.76) and spiritual growth (25.54±5.25). The lowest score was for physical activity (15.68 ±4.67).

**Table 3:** HPLP II total and subscale mean scores

Variables	Mean	SD	Range	Minimum and maximum possible score
HPLP II Total	126.92	19.46	81-171	52-208
Health Responsibility	19.86	4.47	10-34	9-36
Physical activity	15.6	4.58	8-32	8-32
Nutrition	20.2	4.17	11-33	9-36
Spiritual	25.54	5.25	12-35	9-36
IPR	25.73	4.76	13-36	9-36
Stress management	20.0	4.19	12-40	8-32

Association of socio-demographic variables with health promoting lifestyle and social media use*.*Table 4 showed that the BSMAS score was significantly higher among males than females and students who were residing with family, had more health responsibility than those who stayed in hostel. Education was significantly associated with total HPLP-II score as well as Health responsibility, spiritual and inter personal relationship domain.

**Table 4:** Association of socio-demographic variables with health promoting lifestyle and social media use

Gender	Gender	Gender	Gender	Gender
Variables / Categories	Mean Rank	Mean Rank	Z-value	***p*-value**
Variables / Categories	**Male (*n*=17)**	**Female (*n*=108)**	Z-value	***p*-value**
HPLP II Total	74.26	61.23	-1.380	0.168
Health Responsibility	77.97	60.64	-1.840	0.066
Physical activity	81.56	60.08	-2.279	0.023
Nutrition	72.59	61.49	-1.178	0.239
Spiritual	70.68	61.79	-.942	0.346
IPR	62.97	63.00	-.004	0.997
Stress management	60.62	63.38	-.293	0.770
BSMAS Total	85.62	58.83	-2.863	0.004
Residence	Residence	Residence	Residence	Residence
Variables / Categories	Mean Rank	Mean Rank	Z-value	***p*-value**
Variables / Categories	**Family (*n*=38)**	**Hostel (*n*=87)**	Z-value	***p*-value**
HPLP II Total	68.08	60.78	-1.036	0.300
Health Responsibility	73.05	58.61	-2.058	0.040
Physical activity	63.28	62.88	-.057	0.955
Nutrition	72.01	59.06	-1.844	0.065
Spiritual	60.04	64.29	-.605	0.545
IPR	62.49	63.22	-.105	0.916
Stress management	70.11	59.90	-1.455	0.146
BSMAS Total	65.96	60.97	-.715	0.475
Education	Education	Education	Education	Education
Variables / Categories	Mean Rank	Mean Rank	Z-value	***p*-value**
Variables / Categories	**UG (*n*=89)**	**PG (*n*=36)**	Z-value	***p*-value**
HPLP II Total	56.96	77.93	-2.931	0.003
Health Responsibility	58.54	74.03	-2.172	0.030
Physical activity	61.30	67.21	-.828	0.407
Nutrition	59.48	71.71	-1.715	0.086
Spiritual	56.47	79.15	-3.176	0.001
IPR	57.53	76.53	-2.662	0.008
Stress management	59.70	71.17	-1.609	0.108
BSMAS Total	62.19	63.25	-.149	0.882

## Discussion

The present study provides preliminary findings on influence of gender, education, place of residence and substance use among nursing 125 students on their social media use and health promoting lifestyle. The study documents characteristics of the study subjects (Table 1) and current internet and social media use as well as its impact on sleep and other offline activities. Majority of the participants were female (86.4%) which reiterates the fact that nursing in India is a female leading profession where in many states till now boys are not eligible for pursuing this profession. The study sample comprised 71% of undergraduate nursing students as well as 93% who were single and 70% were staying in hostel. This indicates that the nursing course is mainly a residential course. Though substance use is less, still it cannot be ignored as 4% of the sample had history of substance use. The study also documents that health promoting lifestyle is inadequately followed by nursing students (Table 3) and 9% of them were using social media more who might develop problematic use later. Male students had significantly higher BSMAS score and students who were residing with family, had more health responsibility than those who stayed in hostel. Significant association was found between education with total HPLP-II score as well as health responsibility, spiritual, and inter personal relationship domain.

Mean age of access to internet was 16.8 years indicating the adolescents to be the most vulnerable group of excessive internet use. Use of internet for recreation purpose was found to be extreme for few participants. Being on internet and scrolling over social media for 14 hours per day indicates continuous unhealthy over-involvement on social media. A significant portion of the participants had frequent delay in sleeping because of internet as well as social media use. Previous studies from India and abroad also support the current study findings.(3,31) A study conducted in southern India among employees (*n*=250) of various organizations, who were using internet for minimum a year, found that overall delay for going to the sleep was 1.6 hours for the sample.(31) Another exploratory survey conducted among nurse educator students (*n*=49) in Finland, reported daily usage of social media for many hours among nursing and other professional students.(3)

The present study finding showed that mean score of HPLP-II was 126.92, which indicated that students were at a moderate level of health- promoting lifestyle. Regarding the subscale scores, it was observed that the highest scores were for inter personal relationship and spiritual growth whereas physical activity had lowest score followed by health responsibility, stress management and nutrition. Prior studies are also in line with the current study findings.(22,29,32) A study conducted among undergraduate nursing students (*n*=167) in Jordan, found that mean score of total HPLP-II was 127.24±21.03 and spiritual growth had highest scores and lowest in physical activity.(22) Similarly, the research results from a study conducted in China found moderate level of health-promoting lifestyle practices among its students and they scored lowest in exercise and highest in interpersonal relations.(29) The findings are also supported by a study from India (*n*=124) where food practices and physical activity had lower scores.(16) This may be attributed either to their negligence or giving less priority to health because of their extensive theoretical and clinical schedule. Besides, residing in hostel always gives limited options for healthy food practices rather they prefer to go for fast food and other unhealthy choices. Similarly, the fact also supports another finding that the students living with family are more responsible for their health which reiterates the trend of students being away from home or living alone in the hostel and neglecting their health for lack of adult guidance.

Gender was found to be associated with mean HPLP-II score, as reflected by the girls scoring higher than the boys. Boys practiced better physical activity than the girls; while in the remaining five dimensions, there was no significant difference between the genders. Mean score of BSMAS (12.79±5.4) was alarming as the scores ranged from 6 to as high as 30. The findings of association shows that the BSMAS score was significantly associated with gender. It was consistent with previous study findings from Norway (*n*=1,018) where male employees used more social networking site during working hours than females.(33) Another study conducted among Turkish university students (*n*=448) found similar results using different measures.(34) However, both the studies were conducted in the working places. 

Education was found to be significantly associated with total HPLP-II score and subscales of health responsibility, spiritual, and inter personal relationship (Table 4). The findings that health responsibility, spiritual bases and inter personal relationship are more among post graduate students than the undergraduate students may be attributed to the influence of higher education. Undergraduate nursing students might not be aware of the health promoting lifestyle; higher the level of maturity, higher the level of coping and thus healthy coping may lead to healthy lifestyle adaptation. Majority of the participants (88%) pursued some kind of hobby or like to indulge in some offline activities. Online activities should be replaced by offline healthy activities. Students are the future citizens in any country. Their health (both mental & physical) can contribute to their ability to make a difference in society in the future. Hence, health and school personnel need to pay attention. 

Health-promoting lifestyle among nursing students has been investigated by number of studies abroad.(20-22) However, in developing countries like India, health promotion is an important area of discussion as it plays crucial role in maintaining health. Adolescents and youth may neglect their health and give less priority to health promoting lifestyle. Coupled with recent addition of excessive technology use, they are more vulnerable to an unhealthy lifestyle. Though this area is receiving an increasing attention in research, still published data is limited. If an individual practices health-promoting behaviour, she or he is more likely to be healthy while avoiding diseases. Social media provides a common platform for engaging the public worldwide.(12) Though it has promised to have some outstanding effects in reaching the unreached population globally, for majority in their day to day life it brings some negative effects when not used in a healthier way. Data on health promoting lifestyle and social media use may help in finding some better way forward for the affected students. 

Limitations. The present study was not an exception to certain limitations such as cross-sectional descriptive study and small sample size. As this study was only conducted with Indian nursing students, generalization of the findings in other countries has to be cautiously considered. However, there is a need for a larger and randomly selected sample to validate the study findings. The present work has implications based on obtained findings. There is a need to work on psycho-educative module for health promotion activities especially sleep hygiene, social media use and lifestyle change for nursing students. The present study did not explore the process of development of excessive to addictive use of social media, though it gave information about association of social media with sleep, physical activity and students’ voice about their perception regarding use of social media on health promotion. 

Further research. The present study findings highlight the health promoting behaviour of a certain group that belongs to the young adult group and effect of a new trend of social media on it. Similar study among adolescents and adult group as well as some age and culture specific interventional study could be an eye opener for health promotion research. 

## Conclusion.

Use of social media has become an indispensable part of everyday life amongst students. The findings showed the presence of excessive use of social media among male students. The social media was used as a modality to overcome personal problems. Its dysfunctional impact on academics was observed too. This study also revealed the lower scores of health promoting life style domains especially for physical activity, health responsibility, stress management and nutrition. Thus, the findings can be used to enhance social media literacy as well as for promotion of positive life style among nursing students. 
